# Temperature-dependent charge-carrier transport between Si-δ-doped layers and AlGaAs/InGaAs/AlGaAs quantum well with various space layer thicknesses measured by Hall-effect analysis

**DOI:** 10.1038/s41598-020-69153-1

**Published:** 2020-07-27

**Authors:** Wilson Yeung-Sy Su, Victor Chien-Pin Lu, Chii-Bin Wu, Jyh-Shyang Wang, Ji-Lin Shen, Kuan-Cheng Chiu

**Affiliations:** 1 Department of Physics and Center for Nanotechnology, Chung Yuan Christian University, Chung-Li District, Taoyuan, 32023 Taiwan; 2 Conary Enterprise CO., LTD, Industry Accelerator and Incubation Center, Chung Yuan Christian University, Chung-Li District, Taoyuan, 32023 Taiwan

**Keywords:** Condensed-matter physics, Electronics, photonics and device physics, Nanoscale devices

## Abstract

Temperature (*T* = 40 ~ 300 K) dependence of Hall-effect analysis on the dual Si-δ-doped AlGaAs/InGaAs/AlGaAs quantum-well (QW) structures with various space layer thicknesses (*t*_*S*_ = 5, 10 and 15 nm) was performed. An interesting hysteresis behavior of electron sheet concentration [*n*_*2D*_(*T*)] was observed for *t*_*S*_ = 10 and 15 nm but not for *t*_*S*_ = 5 nm. A model involving two different activation barriers encountered respectively by electrons in the active QW and by electrons in the δ-doped layers is proposed to account for the hysteresis behavior. However, for small enough *t*_*S*_ (= 5 nm ≤ 2.5* s*, where *s* = 2.0 nm is the standard deviation of the Gaussian fit to the Si-δ-doped profile), the distribution of Si dopants near active QW acted as a specific form of “modulation doping” and can not be regarded as an ideal δ-doping. These Si dopants nearby the active QW effectively increase the magnitude of *n*_*2D*_, and hence no hysteresis curve was observed. Finally, effects from *t*_*S*_ on the *T*-dependence of electron mobility in active QW channel are also discussed.

## Introduction

**Figure 1:**
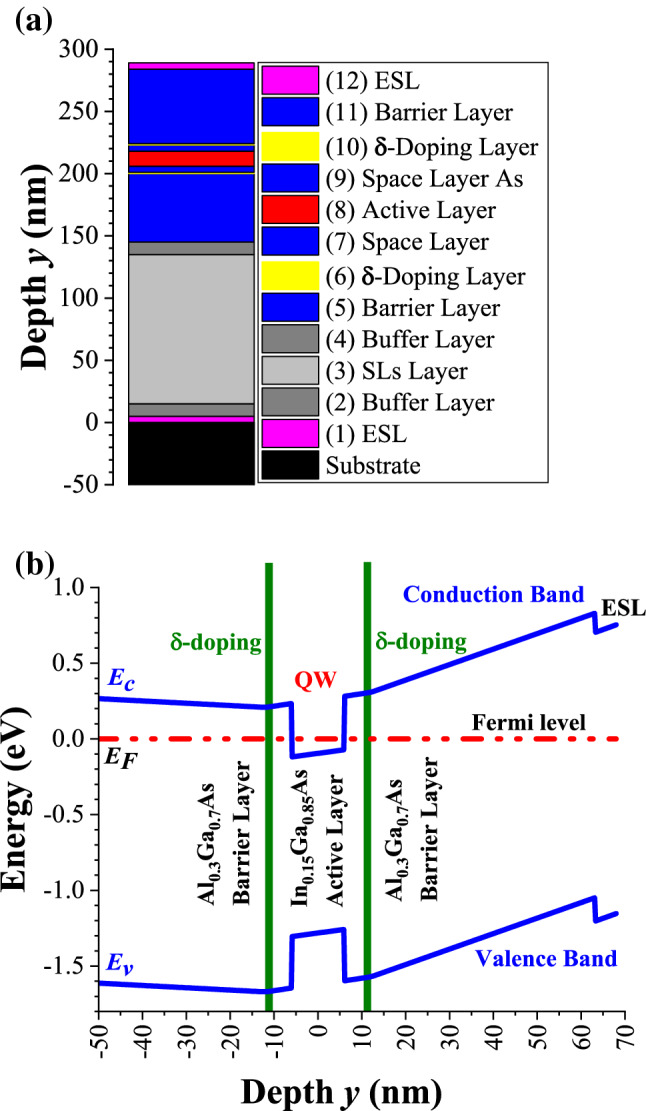
(**a**) Cross-sectional view of the Si-δ-doped QW structure adopted in this work. (**b**) Schematic band diagram nearby QW of the structure.

Motivated by the progress of artificial intelligence and internet of things (AIoT), an intense effort has been devoted to the fabrication and characterization of sensors and actuators to quickly detect and response the variations in the physical world. Among them, the Hall magnetic sensors fabricated from semiconductors are mostly used in contact-less sensors for linear and angular position, velocity and angular frequency, electrical current, etc., and play an important role in the era of AIoT^1^. In particular, the two-dimensional electron gas (2DEG) in Si-δ-doped III–V quantum-well (QW) channel possesses high electron mobility (*μ*_*n*_), high thermal stability, and low noise, and hence is widely adopted as the active layer for high speed and high sensitivity electronic devices^2–6^. In contrast to the homogeneous bulk-doped structure^7–9^, the uniform modulation doping in barrier layers of III–V QW structure can supply charge carriers in the undoped active channel with high mobility due to less scattering from ionized dopants^10^. Furthermore, the single Si-δ-doping (modulation doping with single Si-doping plane)^11–16^ in the barrier layer with appropriate space layer thickness (*t*_*S*_) can offer effectively more 2DEG than the uniform modulation doping. As compared to the single Si-δ-doping, the dual and symmetric Si-δ-doping (modulation doping with two planes of Si evenly separating into two sides of the active channel with appropriate *t*_*S*_, see Fig. 1) can afford nearly a similar electron concentration in the active channel with low induced internal electric field due to the symmetric distribution of Si-δ-dopants and hence provide an even higher *μ*_*n*_ due to the further reduction of scattering effects^17–19^.

For Si-doped Al_*x*′_Ga_1−*x*′_As fabricated by metal organic chemical-vapor deposition (MOCVD) processes under As-rich environments, the Si dopants (with concentration *N*_*Si*_) usually occupy the group III sites^20^ and act either as shallow donors (*N*_*SD*_, with donor level *E*_*SD*_ = *E*_*C*_ – 5.8 meV) for normal substitution or as deep DX centers (*N*_*DD*_, with *E*_*DD*_ = *E*_*C*_ – 145 meV) for broken-bond configurations^12,21–24^, where *E*_*C*_ is the conduction band edge. Assume that *N*_*SD*_ + *N*_*DD*_ = *N*_*Si*_, the ratio of *N*_*DD*_/*N*_*Si*_ changes with Al mole fraction *x*^′^: for *x*^′^ < 0.20, *N*_*DD*_/*N*_*Si*_ = 0; and for 0.20 < *x*^′^ < 0.40, *N*_*DD*_/*N*_*Si*_ increases continuously with *x*^′^^25^. Although this relation is deduced from a homogeneous Si-bulk-doped structure, it still holds in the Si-δ-doping layer due to the similar occupation behaviors of Si atoms in the group III sites as long as the concentration of Si-dopants is below degenerate doping. To achieve a good confinement of 2DEG in the active channel, *x*^′^ = 0.3 is commonly chosen. Accordingly, with high enough temperature, the thermal activation of charge carriers released from DX centers and the transport of carriers across space layer between Si-δ-doped layer and active QW layer should be carefully characterized because both of them play a crucial role in the performance of the devices fabricated.

As depicted in Fig. 1, an undoped In_0.15_Ga_0.85_As was chosen as the active layer because it possesses a higher *μ*_*n*_ than GaAs. In InGaAs system, the increase of indium percentage will enhance *μ*_*n*_, but it also enhances the lattice constant and reduces the bandgap. The enhancement of lattice constant in InGaAs increase the lattice mismatch between InGaAs active layer and AlGaAs barrier layer which will deteriorate the quality of the sandwiched active layer. The reduction of bandgap can induce the noise from thermally generated electron–hole pairs as the device is operating at high temperature. Based on the published data^26^, a compromise of 15% was chosen.

**Figure 2:**
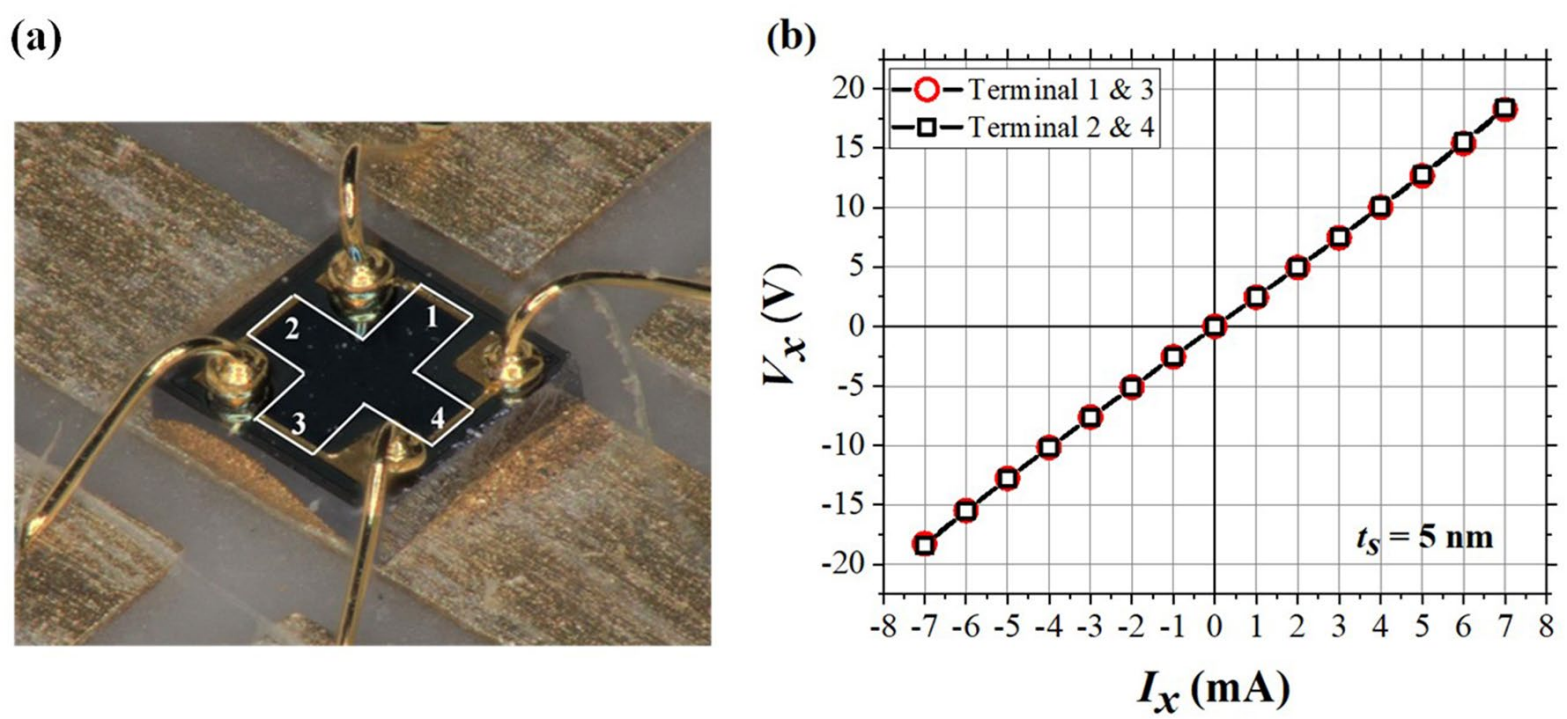
(**a**) Configuration of the cross-like micro-Hall element. (**b**) *I–V* characteristics for a micro-Hall sample with *t*_*S*_ = 5 nm.

Here, a dual and symmetric Si-δ-doped Al_0.3_Ga_0.7_As/In_0.15_Ga_0.85_As/Al_0.3_Ga_0.7_As QW structure as depicted in Fig. 1 was fabricated to be used as the core-element in the micro-Hall magnetic sensors as shown in Fig. 2. The values of *t*_*S*_ = 5, 10, and 15 nm were chosen. The temperature (*T* = 40 ~ 300 K) dependent Hall-effect analyses were conducted, and the electron sheet concentration *n*_*2D*_(*T*) and electron mobility *μ*_*n*_(*T*) in the active channel were deduced. Then, the *T*-dependent charge carriers transport across the space layer together with the effects of *t*_*S*_ on *n*_*2D*_(*T*) and *μ*_*n*_(*T*) are discussed.

## Results and discussion

### Dynamic SIMS measurement

**Figure 3:**
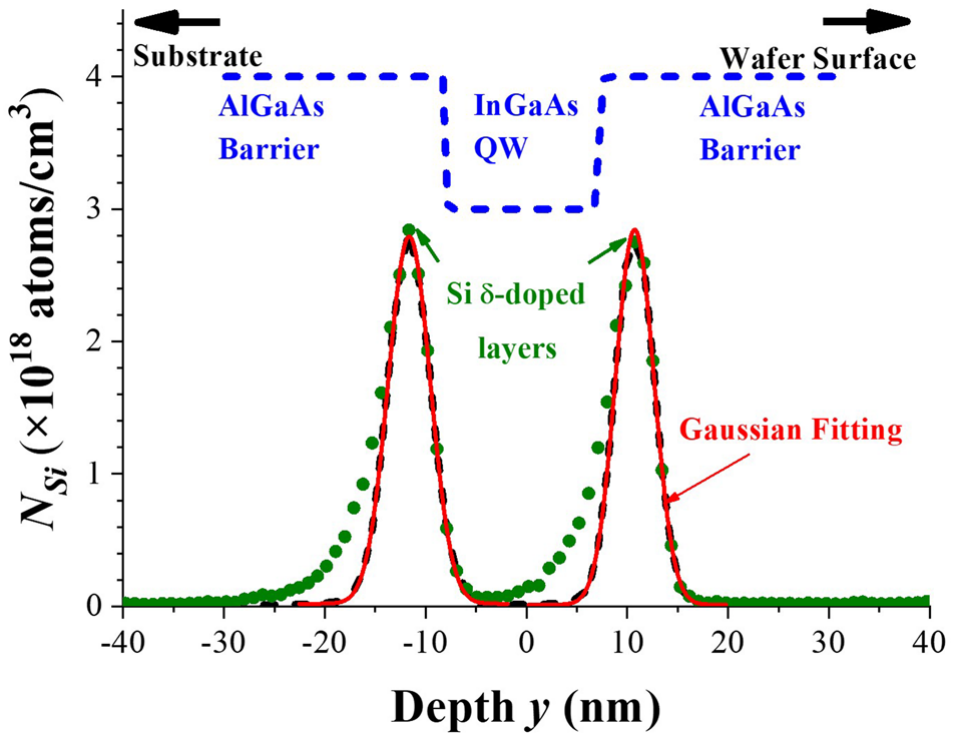
*N*_*Si*_(*y*) measured from dynamic SIMS for a dual Si-δ-doping profile with *t*_*S*_ = 5 nm.

The Si-δ-doping in both sides of active layer provides the 2DEG in active channel, so at first the characteristics of the doping profile *N*_*Si*_(*y*) in-depth distribution for sample with *t*_*S*_ = 5 nm was checked by dynamic secondary ion mass spectrometer (SIMS). As shown in Fig. 3, the asymmetric distribution of *N*_*Si*_(*y*) with respect to the center of each doping profile (at *y*_*δ*_ =  ± 11 nm) can be understood as that the thermal energy caused by the primary ions bombardment could drive some in situ Si dopants to diffuse back. Therefore, an extra tiny residues were added to the falling edge of the δ-doping profile as compared to the leading edge. Therefore, by taking the leading edge into account only, one of the Si-δ-doping profiles as depicted in Fig. 3 can be modeled as a Gaussian distribution^27^ with short enough standard deviation *s*,

1 $$N_{Si} \left( y \right) \, = \, (N_{2D} /({\text2{\pi}}s^{{2}} )^{{{1}/{2}}} ) \times {\exp}[ - (y - y_{\delta} )/s)^{{2}} /{2}] \, + N_{0} ,$$

where *N*_*2D*_ is the Si sheet concentration and *N*_*0*_ is the background. From a least squared fit to these dual Si-δ-doping profiles, the averaged values of *N*_*2D*_ = (1.40 ± 0.07) × 10^12^ cm^−2^ and *s* = 2.00 ± 0.13 nm were obtained, and the two fitted Gaussian profiles were rather symmetric. The total sheet concentration of Si atoms from these dual δ-doping layers was equal to 2 × *N*_*2D*_ = (2.80 ± 0.14) × 10^12^ cm^−2^ and this parameter was held as a constant for the samples fabricated with various *t*_*S*_ in this work. Although the magnitude of *s* = 2 nm was small enough for *t*_*S*_ = 10 and 15 nm (i.e., *t*_*S*_ ≥ 5* s*) such that *N*_*Si*_(*y*) can be reasonably regarded as a δ-doping profile, but for *t*_*S*_ = 5 nm (≤ 2.5* s*) the actual distribution of *N*_*Si*_(*y*) as depicted in Fig. 3 in the narrow regions close to active channel (for *y* = − 11 to − 6 nm and 6 to 11 nm) as well as the tiny amount of Si atoms penetrated into the active region (from *y* = − 6 to + 6 nm) cannot be neglected and their effects on the magnitude of *n*_*2D*_ and on the transport properties will be addressed later.

### Electron sheet concentrations ***n***_***2D***_(***T***) from Hall-effect analysis

**Figure 4:**
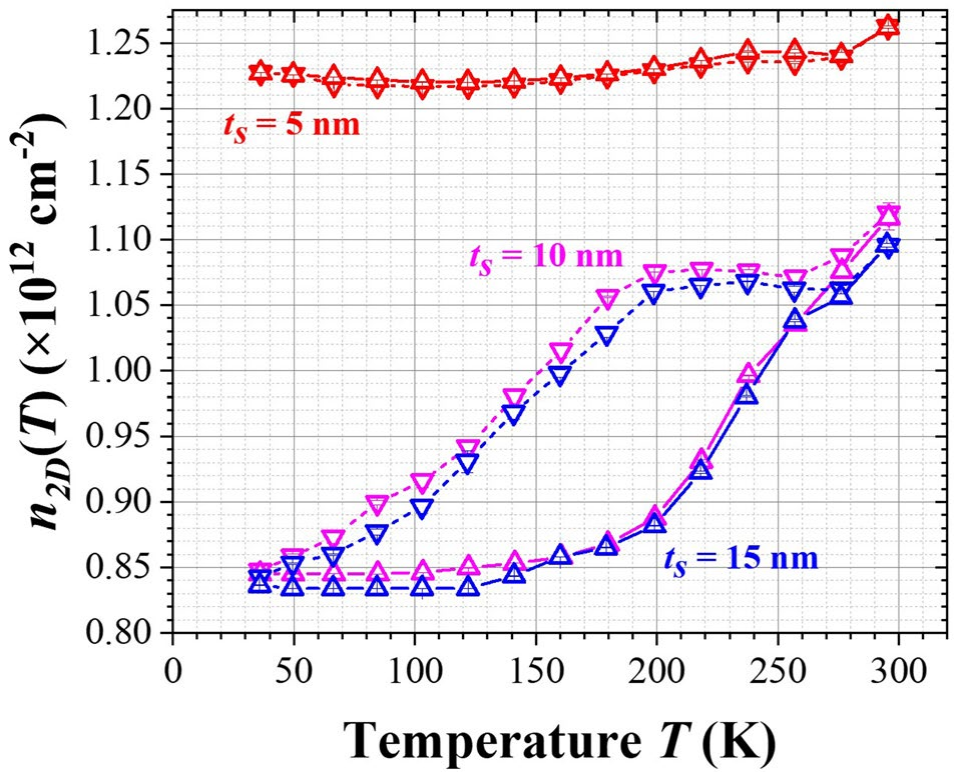
*n*_*2D*_(*T*) measured from Hall-effect analysis for samples with *t*_*S*_ = 5, 10, and 15 nm under decreasing- (▽) and then increasing-*T* (▲) measurements. The magnitude of the error-bar estimated from the measurement is less than the size of the symbol.

Three types of samples are focused in this study with similar structure as Fig. 1a and identical Si-δ-doping profile as Fig. 3 except *t*_*S*_ varied from 5, 10, to 15 nm. The electron sheet concentrations *n*_*2D*_ (= *I*_*x*_*B*_*y*_/*qV*_*H*_) of these samples measured from Hall-effect analysis with decreasing- and then increasing-*T* modes (for *T* between 40 and 300 K) are depicted in Fig. 4. For *t*_*S*_ = 5 nm, *n*_*2D*_(*T*) varied slightly with *T*, but for *t*_*S*_ = 10 and 15 nm, *n*_*2D*_(*T*) varied drastically and exhibited an interesting hysteresis behavior. Besides, at 40 K, *n*_*2D*_ for *t*_*S*_ = 5 nm was 46% higher than *n*_*2D*_ for *t*_*S*_ = 10 and 15 nm. To explain the hysteresis behavior of *n*_*2D*_(*T*) and the effects of *t*_*S*_ on *n*_*2D*_(*T*) and *μ*_*n*_(*T*), the following arguments are proposed.

### Shallow donors versus deep DX centers

In our samples, the AlGaAs barrier layers (except at the δ-doping layers) and InGaAs active channel were undoped, the surface states were passivated by InGaP, and the intrinsic carrier concentrations from these III–V compounds were small enough to be neglected, so the measured *n*_*2D*_(*T*) was assumed dominantly from the dual Si-δ-doping layers. The Si dopants (*N*_*Si*_) in AlGaAs barrier layer can act either as shallow donors (*N*_*SD*_ with *E*_*SD*_ = *E*_*C*_ – 5.8 meV) or as deep DX centers (*N*_*DD*_ with *E*_*DD*_ = *E*_*C*_ – 145 meV)^22^. Based on the model mentioned above^25^, the ratios of *N*_*SD*_/*N*_*Si*_ = 0.3 and *N*_*DD*_/*N*_*Si*_ = 0.7 were calculated for Al_0.3_Ga_0.7_As. As shown in Fig. 4 and from the Gaussian fit, the total sheet concentration of Si atoms from dual δ-doping layers was 2 × *N*_*2D*_. Thus, 30% of the Si dopants (acted as shallow dopants) became nearly completely ionized at *T* = 300 K, and contributed a sheet electron density *n*_*SD*_ ~ 0.3 × 2 × *N*_*2D*_ = (0.84 ± 0.04) × 10^12^ cm^−2^. As depicted in Fig. 4, this value coincided with the lowest value of *n*_*2D*_ observed for samples with *t*_*S*_ = 10 and 15 nm at *T* = 40 K, i.e., in such situations *n*_*2D*_ was composed of the electrons completely ionized from shallow donors (*n*_*SD*_). Therefore, for *t*_*S*_ = 10 and 15 nm, the measured values of *n*_*2D*_ > *n*_*SD*_ at high *T* suggests that electrons partially ionized from the DX centers (*n*_*DD*_) must be taken into account, which gives *n*_*DD*_ = *n*_*2D*_ − *n*_*SD*_. From the ratio of *n*_*DD*_/(0.7 × 2 × *N*_*2D*_), which equals the unoccupied probability [1 − *f*(*T, E*_*DD*_)] of electrons at *E*_*DD*_, then the occupied probability for electrons at DX centers, *f*(*T*, *E*_*DD*_), can be evaluated from Fermi–Dirac statistics^28^ with

2 $$f\left( {T,E_{DD} } \right) \, = { 1}/\left[ {{1 } + {\exp}\left( {\left( {E_{DD} - E_{F} } \right)/kT} \right)} \right],$$

where *E*_*F*_ is the Fermi energy level and *kT* is the thermal energy. Accordingly, the energy difference of *E*_*DD*_ – *E*_*F*_ can also be estimated. For samples with various *t*_*S*_, the associated parameters (*n*_*2D*_, *n*_*SD*_, *n*_*DD*_, *f*(*E*_*DD*_), and *E*_*DD*_ – *E*_*F*_) are listed in Table 1 for *T* = 300 K.

**Table 1:** Associated parameters for evaluating *E*_*DD*_ – *E*_*F*_ at each Si-δ-doping layer and Δ*V* across *t*_*S*_ for samples with various *t*_*S*_ at 300 K.

*t*_*S*_ (nm)	*n*_*2D*_/2 (10^12^ cm^−2^)	*n*_*SD*_/2 (10^12^ cm^−2^)	*n*_*DD*_/2 (10^12^ cm^−2^)	*f*(*E*_*DD*_)	*E*_*DD*_—*E*_*F*_ (meV)	*F* (10^4^ V/cm)	Δ*V* (mV)
5	0.631 ± 0.001	0.42 ± 0.02	0.21 ± 0.02	78.4 ± 1.6%	− 33 ± 2	9.32 ± 0.01	47
10	0.559 ± 0.005	0.42 ± 0.02	0.14 ± 0.02	85.6 ± 2.7%	− 45 ± 6	8.27 ± 0.07	83
15	0.548 ± 0.001	0.42 ± 0.02	0.12 ± 0.02	87.6 ± 2.7%	− 50 ± 6	8.09 ± 0.01	121

The total sheet concentration of Si atoms from dual δ-doping layers was fixed at (2.80 ± 0.14) × 10^12^ cm^−2^.

Note that *n*_*2D*_ was taken from Hall-effect analysis (see Fig. 4), *n*_*SD*_ was taken from SIMS analysis and estimated by information based on Ref.^25^, and *n*_*DD*_ = *n*_*2D*_ − *n*_*SD*_*.*

### Simplified band diagrams modelling by two back-to-back capacitors

**Figure 5:**
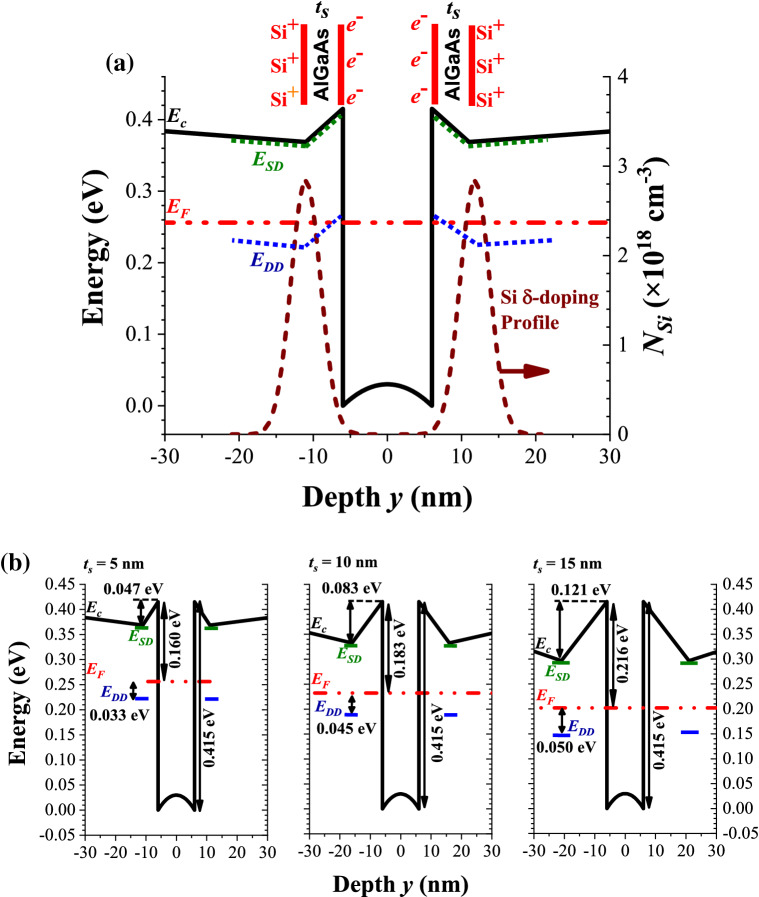
(**a**) Simplified conduction band diagram deduced from the dual Si-δ-doping layers based on two back-to-back capacitors at 300 K. (**b**) Comparison of the corresponding band diagrams for samples with *t*_*S*_ = 5, 10, and 15 nm.

Unlike homogeneous bulk-doped materials, where the electrons and ionized donors appear in the same spatial location, for Si-δ-doped heterojunction QW the electrons are transferred to the QW region while the ionized donors remain in the δ-doped layers^11–19^. Because of the separation of charges, a model consisted of two back-to-back capacitors was chosen to calculate the internal transverse electric field (*F*) and electric potential difference Δ*V* across the space layer. Furthermore, a symmetric band diagram was assumed for simplicity for a dual and symmetric δ-doped layers^17–19^ as depicted in Fig. 5a. In such a case, the capacitor has a positive surface charge density + *σ* (= *q* × (*n*_*SD*_ + *n*_*DD*_)/2) from ionized Si donors located at one of the δ-doping layers and a negative surface charge density − *σ* (= − *q* × *n*_*2D*_/2) accumulated by electrons at one edge of the active QW channel, and *n*_*2D*_ = *n*_*SD*_ + *n*_*DD*_ is assumed as mentioned above. Accordingly, the values of *F* within the capacitor (= *σ*/*ε*_*0*_*ε*_*r*_, where the dielectric constant *ε*_*r*_ = 12.24 is taken for Al_0.3_Ga_0.7_As^29^) and Δ*V* across *t*_*S*_ were calculated for *t*_*S*_ = 5, 10, and 15 nm, separately. These data at *T* = 300 K are also listed in Table 1. By taking the conduction band offset of 0.415 eV for the Al_0.3_Ga_0.7_As/In_0.15_Ga_0.85_As QW heterojunction^30^, the simplified conduction band diagrams for various *t*_*S*_ are depicted as Fig. 5b. Based on these band diagrams derived from this simplified scheme, *E*_*F*_ locates reasonably far away from *E*_*C*_ at the Si-δ-doped layer, which suggests that the conductivity was dominantly by the 2DEG in the active channel for our samples with moderate Si-dopants (*N*_*2D*_ = 1.40 × 10^12^ cm^−2^). Only for very high Si-doping such that *E*_*F*_ is very close to *E*_*C*_, the parallel conductivity over the doped layers should be taken into account.

### Origin of hysteresis curves of ***n***_***2D***_(***T***) for ***t***_***S***_ = 10 and 15 nm

**Figure 6:**
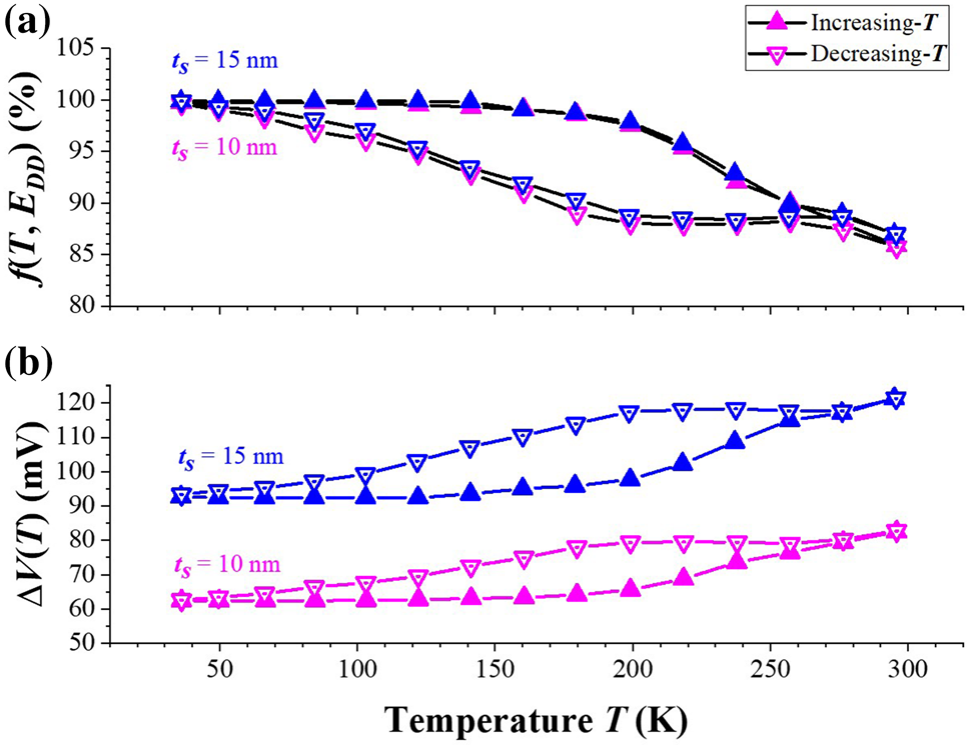
(**a**) *f*(*T*, *E*_*DD*_) and (**b**) Δ*V*(*T*) evaluated for Hall samples with *t*_*S*_ = 10 and 15 nm.

As demonstrated in Fig. 5, the hysteresis curves of *n*_*2D*_(*T*) observed for *t*_*S*_ = 10 and 15 nm in Fig. 4 could be understood as followings. In general, if electrons generated and recaptured from their donor states involve only one activation energy (as in the uniform homogeneous bulk-doped sample) and if the relaxation time is short enough, then as long as the thermal equilibrium is nearly reached, no hysteresis curve will be observed for decreasing- and increasing-*T* measurements. In this work, the time for the system stayed at each *T* before Hall-effect measurement was much larger than the relaxation time expected. Therefore, more than one activation energy associated with the charge carriers transfer were required to account for the hysteresis curves observed in Fig. 4. During a decreasing-*T* measurement from 300 to 280 K, the electrons in active QW channel (with energy around *E*_*F*_) even though encounter a large activation barrier (*E*_*A1*_ = 216 meV for *t*_*S*_ = 15 nm as depicted in Fig. 5b) to return the donor states in Si-δ-doped layer, with a thermally assisted tunneling the transfer of electrons from active QW channel to the δ-doping layer can be rather efficient and then *n*_*2D*_(*T*) decreased with decreasing *T* (i.e., more DX levels become occupied, the probability of *f*(*T*, *E*_*DD*_) increased and Δ*V*(*T*) decreased). However, as *T* decreased from 280 to 200 K, with less thermally assisted tunneling the excess electrons are then persistently confined in the active 2D channel, *n*_*2D*_(*T*) and the corresponding *f*(*T*, *E*_*DD*_) and Δ*V*(*T*) all remained constant as shown in Fig. 6. As *T* further decreased from 200 to 40 K, the value of *F* induced by the non-equilibrium extra space-charges would now assist the electrons transfer from active channel to δ-doped layers, thus *n*_*2D*_(*T*) dropped again and the occupied probability of DX levels, *i.e*., *f*(*T*, *E*_*DD*_), started to increase and Δ*V*(*T*) across the capacitor reduced. At *T* = 40 K, *f*(*T*, *E*_*DD*_) reached nearly 100%, and in this situation *n*_*2D*_ was totally dominated by the completely ionized shallow donors (*n*_*SD*_). On the other hand, during an increasing-*T* measurement from 40 to 150 K, the electrons thermally released from DX centers in Si-δ-doping layer were rather small as compared with *n*_*SD*_ and thus *n*_*2D*_ was very close to *n*_*SD*_ (see Fig. 4), hence *f*(*T*, *E*_*DD*_) remained nearly 100% and Δ*V*(*T*) kept as a constant (see Fig. 6). For *T* increased from 150 to 280 K, the thermally released electrons from DX centers to conduction band in AlGaAs become noticeably and by a longitudinal applied bias these electrons then transferred to the active channel efficiently due to a small activation barrier (*E*_*A2*_ = *q*Δ*V* = 95 ~ 117 meV for *t*_*S*_ = 15 nm at *T* = 150 ~ 280 K, see Fig. 6b). Therefore, *n*_*2D*_(*T*) and Δ*V*(*T*) increased while *f*(*T*, *E*_*DD*_) decreased with increasing *T*. Because of these different activation barriers encountered with respect to decreasing- and increasing-*T* modes (*E*_*A1*_ versus *E*_*A2*_), a *T*-dependent hysteresis on *n*_*2D*_(*T*) was resulted as shown in Fig. 4 for *t*_*S*_ = 10 and 15 nm. Besides, based on the model of two back-to-back capacitors, the values of *F* for these two samples were nearly alike due to the good match of *n*_*2D*_(*T*) as shown in Table 1 and Fig. 4. Hence, as depicted in Fig. 6, *f*(*T*, *E*_*DD*_) followed the same trend while Δ*V*(*T*) (= *F*•*t*_*S*_) acted differently for these two samples. Furthermore, as displayed in Fig. 4, the hysteresis curves ended at *T* ~ 280 K for these two samples. Note that the occupied probability for electron at DX center is related to an energy difference of *E*_*DD*_ − *E*_*F*_. As listed in Table 1, the magnitudes of *E*_*DD*_ − *E*_*F*_ were nearly comparable to *kT* for *T* = 280 ~ 300 K. With the support of thermally assisted tunneling and a longitudinal applied bias, the transfer of electrons through the space layers became reversible*.* Because of the upper limit of our cryostat, no further experimental data are provided for *T* > 300 K.

### Effects from non-ideal Si-δ-doped profiles for ***t***_***S***_ = 5 nm

As also illustrated in Fig. 4, the fact of no hysteresis curve of *n*_*2D*_(*T*) observed for small *t*_*S*_ = 5 nm suggests that the non-ideal Si-δ-doped profiles as shown in Fig. 3 was the culprit. For *t*_*S*_ = 5 nm = 2.5* s*, the actual distribution of *N*_*Si*_(*y*) near active QW (for *y* = − 11 to − 6 nm and 6 to 11 nm in Fig. 3) acted as a specific form of “modulation doping” with strong gradient toward QW channel and could not be regarded as an ideal δ-doping. Meanwhile, the band bending up as illustrated in Fig. 5a can shift *E*_*DD*_ (corresponding to the Si DX levels in Al_0.3_Ga_0.7_As barrier layer with energy of *E*_*C*_ – *E*_*DD*_ = 145 meV) above *E*_*F*_. Therefore, those electrons from DX centers located in the vicinity close to QW with energy around *E*_*F*_ can hop through these DX centers and fall into the QW channel. Based on the data at low enough *T* = 40 K as depicted in Fig. 4, *n*_*2D*_ (≅ 1.23 × 10^12^ cm^−2^) for *t*_*S*_ = 5 nm was higher than *n*_*2D*_ (≅ 0.84 × 10^12^ cm^−2^, dominated by *n*_*SD*_) for *t*_*S*_ = 10 and 15 nm by 46% [= (1.23 − 0.84)/0.84)]. The percentage of the extra electrons released from DX centers in this specific “modulation doping” was estimated around 20% [= (1.23 − 0.84) × 10^12^/(2 × *N*_*2D*_ × 0.7)]. These electrons persistently confined within the active QW channel not only enhanced the total values of *n*_*2D*_ but also compensated the *T*-dependence of *n*_*2D*_ from the typical DX centers as mentioned above. Thus *n*_*2D*_(*T*) for *t*_*S*_ = 5 nm turned out to be relatively weak *T*-dependence with respect to decreasing- and increasing-*T* modes and hence no hysteresis curve was observed.

### Effects of ***t***_***S***_ on the ***T***-dependence of electron mobility

**Figure 7:**
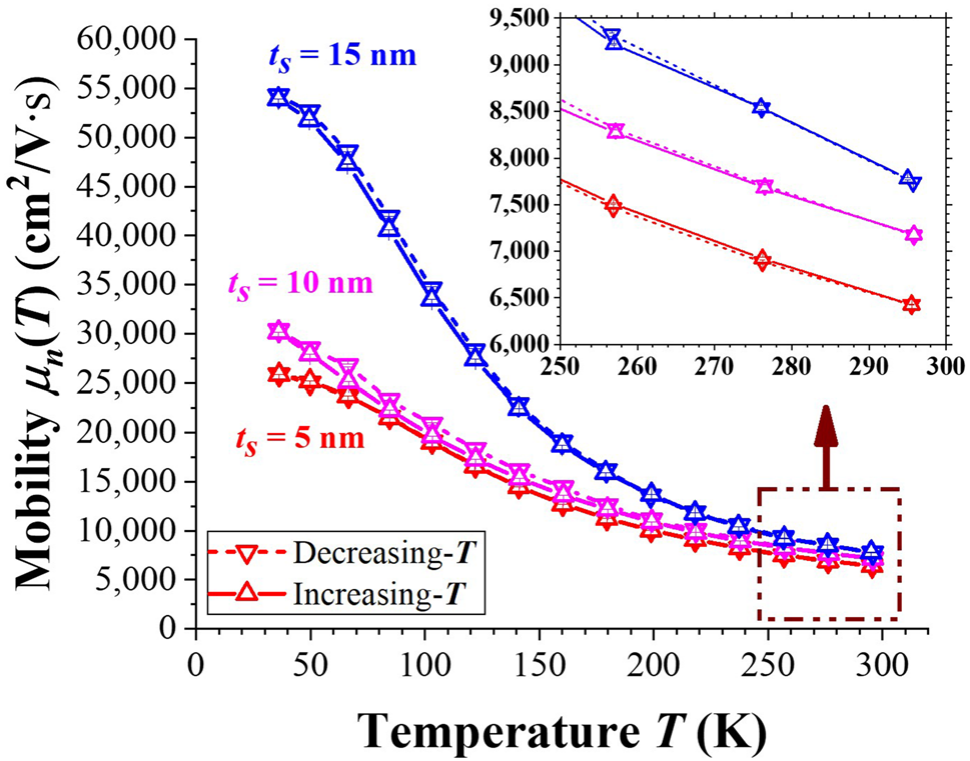
*μ*_*n*_(*T*) measured from Hall-effect analysis for samples with various *t*_*S*_ under decreasing *T* (▽) and then increasing *T* (▲) measurements. The inset enlarges the corresponding values near room temperature. The magnitude of the error-bar estimated from the measurement is less than the size of the symbol.

The *T*-dependence of electron mobility *μ*_*n*_(*T*) deduced from Hall-effect analysis on samples with various *t*_*S*_ (= 5, 10 and 15 nm) is demonstrated in Fig. 7. For *T* = 40 K with less phonon scattering, *μ*_*n*_ is very sensitively dependent on the Coulombic scattering from the ionized Si-donors located at δ-doped layers separated by *t*_*S*_ from the active channel. In addition, the very small amount of Si dopants entered into the In_0.15_Ga_0.85_As active channel (from *y* = − 6 to + 6 nm as shown in Fig. 5a) could further reduce *μ*_*n*_. In viewing of these two factors, the sample with *t*_*S*_ = 15 nm possessed a highest *μ*_*n*_(40 K) = (5.41 ± 0.02) × 10^4^ cm^2^ V^−1^ s^−1^ among these three types of samples. With increasing *T*, *μ*_*n*_ decreases dramatically due to phonon scattering in the active channel. Nevertheless, *μ*_*n*_(300 K) for all dual and symmetric Si-δ-doped AlGaAs/InGaAs/AlGaAs QW samples fabricated by this work still possessed relatively high values of (6.43 ± 0.06) × 10^3^, (7.17 ± 0.16) × 10^3^ and (7.76 ± 0.03) × 10^3^ cm^2^ V^−1^ s^−1^ for *t*_*S*_ = 5, 10 and 15 nm, respectively. A high value of *μ*_*n*_ is one of the important figures of merit for high sensitivity magnetic sensor.

## Conclusion

In this work, dual and symmetric Si-δ-doped AlGaAs/InGaAs/AlGaAs QW structures of various *t*_*S*_ (= 5, 10 and 15 nm) were fabricated and characterized. At first, from a Gaussian fit on the dynamic SIMS data for the dual Si-doping profile, the two Si dopant-profiles were rather symmetric and *N*_*2D*_ = 1.40 × 10^12^ cm^−2^ and *s* = 2.0 nm were obtained for each profile. Next, *n*_*2D*_(*T*) and *μ*_*n*_(*T*) in the active QW channel of these samples were measured from Hall-effect analysis with decreasing- and then increasing-*T* modes. Interesting hysteresis curves of *n*_*2D*_(*T*) were observed for *t*_*S*_ = 10 and 15 nm but not for *t*_*S*_ = 5 nm. Because of the charge separation for the Si-δ-doped AlGaAs/InGaAs/AlGaAs QW structure, a simplified energy-band diagram based on two back-to-back charged capacitors was proposed to explain these phenomena. Due to the different activation barriers encountered respectively by electrons in the active QW and by electrons in the δ-doped layers during decreasing- and then increasing-*T* modes, a hysteresis on *n*_*2D*_(*T*) was obtained for *t*_*S*_ = 10 and 15 nm. Besides, at *T* = 40 K the lowest value of *n*_*2D*_ = 0.84 × 10^12^ cm^−2^ observed for samples with *t*_*S*_ = 10 and 15 nm indicates that in such situations *n*_*2D*_ was composed of the electrons completely ionized from shallow donors (*n*_*SD*_) which agrees well with the results from the proposed model based on experimental SIMS data. However, for small enough *t*_*S*_ = 5 nm (i.e., *t*_*S*_ ≤ 2.5* s*), the actual distribution of *N*_*Si*_(*y*) near QW could not be regarded as an ideal δ-doping. The amount of Si atoms nearby the active QW channel acted as a specific form of “modulation doping” and effectively increased the level of *n*_*2D*_, and hence no hysteresis curve was observed. Finally, the effects of *t*_*S*_ on *μ*_*n*_(*T*) for these three structures were also addressed.

## Methods

### Fabrication of the epi-structure

The epi-layers of the Hall sample on a semi-insulating GaAs substrate depicted in Fig. 1 were fabricated by MOCVD under As-rich environments^20^ in the following steps. (1) At first, 5 nm InGaP and 10 nm GaAs buffer layers were deposited sequentially, followed by 10 pairs of 6 nm AlAs and 6 nm GaAs superlattice layers, and another 10 nm GaAs buffer layer to release the strain resulted from lattice mismatch. (2) After deposition of an undoped 55 nm Al_0.3_Ga_0.7_As barrier layer, the first Si-δ-doping layer with sheet concentration of 1.4 × 10^12^ cm^−2^ was achieved by injection a high SiH_4_ doping flow and followed by an undoped Al_0.3_Ga_0.7_As space layer with thickness *t*_*S*_. (3) The active layer was fabricated by In_0.15_Ga_0.85_As with thickness *t*_*A*_ = 12 nm. (4) After an undoped Al_0.3_Ga_0.7_As space layer with thickness *t*_*S*_, the second symmetric Si-δ-doping layer was repeated, then followed by a 60 nm undoped Al_0.3_Ga_0.7_As barrier layer. (5) Finally, a passivation layer of 5 nm InGaP was deposited on top of the Hall element to reduce the effects from surface states and acted as an etching stopping layer (ESL). For ohmic contact with the active channel, after capped with 15 nm Si-doped GaAs cap layer, the standard ohmic contact were applied by adding a metal layer series of AuGe/Ni/Au^31^ of total thickness 350 nm, thermally driven to make a contact with the QW active channel, and finalized with a Ti/Au bonding pad. The dimensions of the cross-like micro-Hall element as illustrated in Fig. 2 were 420 × 420 μm^2^, with channel width *W* = 115 μm and length *L* = 350 μm. According to the *I–V* characteristics for a micro-Hall sample with *t*_*S*_ = 5 nm as depicted in Fig. 2b, the charge transport properties along the active channels exhibited a good ohmic contact behavior and a nice symmetry.

### Characterization

The characteristics of the Si-δ-doping in-depth distribution were first verified by dynamic mode of secondary ion mass spectroscopy (SIMS, outsourced by EAG Laboratories). Then the Hall-effect analysis was conducted by a Keithley 7065 system^32^ on the cross-like sample with 4 numbered terminals as depicted in Fig. 2, and the linear *I*_*x*_–*V*_*x*_ characteristics in each active channel between terminals 1 ↔ 3 and 2 ↔ 4 were confirmed for *I*_*x*_ < 8 mA, respectively. With a steady current *I*_*x*_ = 1.0 mA applied between terminals 1 ↔ 3 under a perpendicular *B*_*y*_ = 5.0 kG, the Hall voltage across terminals 2 ↔ 4 were consecutively measured for ten times and averaged to give the value of *V*_*H1*_. By reversing the direction of *I*_*x*_, *V*_*H2*_ was obtained similarly. Next, *I*_*x*_ was switched from terminals 1 ↔ 3 to 2 ↔ 4, and by repeating the above procedures *V*_*H3*_ and *V*_*H4*_ across terminals 1 ↔ 3 were obtained again. Finally, by reversing the direction of the *B*-field and following the same procedures, *V*_*H5*_ ~ *V*_*H8*_ were measured correspondingly. From the combination of these eight measurements to compensate the offset voltage due to any asymmetry of the cross-like Hall sample, the Hall coefficient *R*_*H*_ was obtained^24^ and the carrier concentration *n*_*0*_ = 1/*qR*_*H*_ was calculated. Furthermore, for resistivity measurement, the van der Pauw model was adopted with a current applied between terminals 1 and 2 and the voltage measured across terminals 3 and 4. By switching the direction of the current and then rotating the sequence of the contact terminals, again a total of eight measurements were taken separately, and the averaged resistivity *ρ* of the sample was evaluated. From *n*_*0*_ and *ρ*, the averaged value of *μ*_*n*_ in the active 2DEG channel was estimated. The temperature (*T*) of the sample during the Hall-effect analysis was controlled by a cryostat system. The cooling or heating rate was set at 2.0 K min^−1^ for each Δ*T* = 20 K, and then the system stayed at each *T* for 10 min to allow the sample to reach near equilibrium before Hall-effect measurement. The period for one set of Hall-data acquisition at each *T* took another 15 min.
